# The complete genome sequence of a novel heterotrophic nitrification aerobic denitrification bacterium *Stutzerimonas kunmingensis* L4

**DOI:** 10.1128/mra.00229-25

**Published:** 2025-06-12

**Authors:** Kai Liu, Shuangming Sui, Jian Gao, Mingjun Liao

**Affiliations:** 1 Hubei Key Laboratory of Environmental Geotechnology and Ecological Remediation for Lake and River, Hubei University of Technology 47896, Wuhan, China; 2 Key Laboratory of Intelligent Health Perception and Ecological Restoration of Rivers and Lakes, Ministry of Education, Hubei University of Technology 47896, Wuhan, China; The University of Arizona, Tucson, Arizona, USA

**Keywords:** *Stutzerimonas kunmingensis*, nitrogen metabolism, toluene, dioxin

## Abstract

We present the complete genome of *Stutzerimonas kunmingensis* L4, isolated from sewage sludge. The genome consists of a circular chromosome (4,661,724 bp) with a GC content of 62.64%, containing several Heterotrophic Nitrification-Aerobic Denitrification (HNAD) and aromatic compound degradation genes.

## ANNOUNCEMENT

*Stutzerimonas kunmingensis* was first isolated from phosphate mines (1). Subsequently, the pressure-tolerant strain *Stutzerimonas kunmingensis* 7850S was isolated from the sediments of the Mariana Trench (2). Here, we report the complete genome sequence of *Stutzerimonas kunmingensis* L4 isolated from the sewage sludge collected from a sewage treatment plant in Inner Mongolia, China (40.13368°N, 111.42779°E). Approximately 10 g of sludge was homogenized in sterile saline, serially diluted, and then inoculated onto the heterotrophic nitrification medium (HNM) (3). After culturing at 10°C for 72 hours, the colonies were streaked onto HNM agar to obtain pure cultures. A single colony was selected and stored in a solution containing 20% glycerol at −80°C.

Cells were collected by centrifuging the culture in HNM for 10 minutes at 8,000 × *g* after 72 hours of cultivation. Genomic DNA was extracted according to the instructions of the Wizard Genomic DNA Purification Kit (Promega, Madison, WI, USA). The purified genomic DNA was quantified using a TBS-380 fluorometer (Turner BioSystems Inc., Sunnyvale, CA). The complete genome of *Stutzerimonas kunmingensis* L4 was sequenced using a combination of PacBio RSII single-molecule real-time (SMRT) sequencing and the Illumina sequencing platform (4). For Illumina sequencing, at least 1 µg of genomic DNA was fragmented with Covaris, and the library was prepared using the NEXTflex Rapid DNA-Seq Kit (Bioo Scientific, USA). The Illumina library was sequenced on the Illumina NovaSeq 6000 (2 × 150 bp) platform by Majorbio (Shanghai, China). For PacBio sequencing, at least 15 µg of genomic DNA was processed with G-tubes (Covaris, MA) to fragment the genomic DNA into approximately 10 kb fragments. Then, the fragments were purified according to the PacBio instructions (Pacific Biosciences, CA), end-repaired, and ligated with SMRTbell sequencing adapters at both ends. The obtained sequencing library was purified three times using 0.45 times the volume of Agencourt AMPure XP beads (Beckman Coulter Genomics, MA). Then, the single-stranded library was circularized by annealing, bound to the polymerase at the bottom of the fixed zero-mode waveguides, and sequencing reaction reagents were added. After each base pair synthesis, a corresponding light was emitted and detected. Each base synthesis was displayed as a pulse peak, and real-time detection was carried out with a high-resolution optical detection system. PacBio generated 40,148 reads (39,135,442 base pairs), with a sequencing depth of 83.95 (N50: 10,249 bp). Illumina generated 3,886,205 × 2 raw paired-end reads (1,173,633,910 base pairs). The coverage rates of Illumina and PacBio read mapping were 98.95% and 100%, respectively. The Illumina data were trimmed using the fastp software (version 0.20.0) (5). The third-generation sequence assembly was performed using the Unicycler (version 0.4.8) assembly software (6). During the assembly process, the pilonjin software was used for sequence correction. If there was an overlap of a certain length at both ends of the final assembled sequence, the sequence was circularized, and the overlap sequence at one end was truncated to finally obtain the complete chromosomal and plasmid sequences. GeneMarks (version 4.3) was used to predict the coding sequences (CDS) in the genome. In addition, Barrnap (version 0.9), tRNA-SCAN-SE (version 2.0.12) (7), and Infernal (version 1.1.4) were used to predict rRNA, tRNA, and sRNA genes, respectively.

The genome consists of a circular chromosome with no plasmid detected. The complete genome size of strain L4 is 4,661,724 bp, and the GC content is 62.64%. The total number of genes is 4,406, among which the total number of CDS is 4,327, and the genes encoding proteins (i.e., coding genes) are 4,263. The total number of RNA genes is 79, including 4 5S rRNAs, 4 16S rRNAs, and 4 23S rRNAs. There are 63 transfer RNAs (tRNAs), and the number of non-coding RNAs (ncRNAs) is 4. Genome sequence analysis revealed that strain L4 comprises HNAD-associated genes, including *npd*, *nas*AB, *nap*AB, *nir*K, *nir*S, *nor*BC, and *nos*Z. Furthermore, the genome comprises genes associated with the degradation of aromatic compounds, including benzoate, toluene, xylene, and dioxin.

## Data Availability

The genome sequence and raw sequencing reads for strain L4 were deposited under GenBank accession number CP183060, BioProject accession number PRJNA1227209, and BioSample accession number SAMN46975402. The raw reads have been deposited in the Sequence Read Archive (SRA) under SRA accession number SRR32453834.
